# An On-Demand Emergency Packet Transmission Scheme for Wireless Body Area Networks

**DOI:** 10.3390/s151229819

**Published:** 2015-12-04

**Authors:** Moshaddique Al Ameen, Choong Seon Hong

**Affiliations:** Department of Computer Science and Engineering, College of Electronics & Information, Kyung Hee University, Yongin-si, Gyeongggi-do 17104, Korea; ameen@khu.ac.kr

**Keywords:** body area network (BAN), emergency, wake up radio, healthcare, media access control (MAC)

## Abstract

The rapid developments of sensor devices that can actively monitor human activities have given rise to a new field called wireless body area network (BAN). A BAN can manage devices in, on and around the human body. Major requirements of such a network are energy efficiency, long lifetime, low delay, security, *etc.* Traffic in a BAN can be scheduled (normal) or event-driven (emergency). Traditional media access control (MAC) protocols use duty cycling to improve performance. A sleep-wake up cycle is employed to save energy. However, this mechanism lacks features to handle emergency traffic in a prompt and immediate manner. To deliver an emergency packet, a node has to wait until the receiver is awake. It also suffers from overheads, such as idle listening, overhearing and control packet handshakes. An external radio-triggered wake up mechanism is proposed to handle prompt communication. It can reduce the overheads and improve the performance through an on-demand scheme. In this work, we present a simple-to-implement on-demand packet transmission scheme by taking into considerations the requirements of a BAN. The major concern is handling the event-based emergency traffic. The performance analysis of the proposed scheme is presented. The results showed significant improvements in the overall performance of a BAN compared to state-of-the-art protocols in terms of energy consumption, delay and lifetime.

## 1. Introduction

Recent advances in wireless, pervasive and ubiquitous information and communication technologies (ICT) have enabled sensor devices to safely operate on and around the human body. These devices monitor the body functions and the surrounding environment. A network of such devices is known as a body area network (BAN) [1,2]. A BAN has great potential to improve the quality of a personal healthcare system. It can provide cost-effective, portable and reliable healthcare services. The seamless integration of different systems and applications provides improved quality of life [3]. A BAN system aims to deliver healthcare not only to patients in hospitals, but also in workplaces and private areas, such as homes and remote locations. The applications of a BAN [4] cover both medical and non-medical fields.

**Figure 1:**
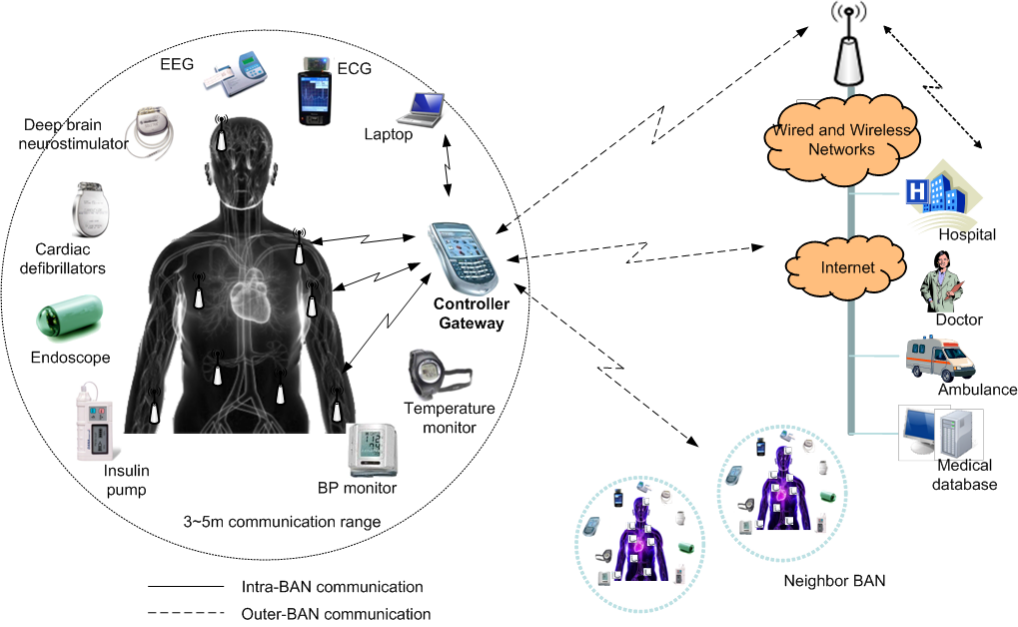
A typical architecture of a BAN.

A typical BAN structure is shown in Figure 1. It consists of devices (also called BAN nodes or BN) and a BAN network coordinator (BNC). The communication in a BAN can be of two types: intra-BAN and outer-BAN. In this work, we focus on intra-BAN communication. A BAN can have normal (scheduled) or emergency traffic (random). BAN devices are usually resource constrained in terms of processing capability and battery and memory capacity. Low energy consumption, delay, lifetime and security are among the key design factors of a BAN.

Energy is consumed for sensing and processing the data and communication. Communication consumes the greatest amount of energy. Efficient energy management is a key requirement in a BAN. A popular method to conserve energy is to switch off (sleep) the transceivers of a node. However, to communicate, the transceivers must be switched on (wake up). This sleep and wake up mechanism is an integral part of energy-efficient operation in a sensor network. Various wake up mechanisms [5] have been proposed to increase the performance of the network. The major approaches are

- Internal self-triggered wake up;
- External out-of-band radio-triggered wake up.

The internal self-triggered wake up is performed by the device itself. In a normal scenario, it uses a timer to trigger the wake up. It is the most widely-used method. It can be scheduled or unscheduled. The external out-of-band radio-triggered wake up is performed by sending an interrupt signal through the pins of the MCU.

In existing systems, if the sleeping node is the intended receiver, the sender must defer transmitting until it is awake. The majority of the state-of-the-art MAC protocols, such as Sensor MAC (S-MAC) [6], Time-out MAC (T–MAC) [7], Berkeley MAC (B-MAC) [8], WiseMAC [9] and X-MAC [10], employ this procedure. These protocols lack a suitable mechanism to immediately communicate if the node is in the sleeping state. This hampers immediate emergency traffic communication. Use of an “always on” sink device is not feasible. There is a high probability that the downlink communication may suffer from a longer delay in an unscheduled environment. This has been observed in the existing IEEE802.15.x standard MAC protocols, as well. To overcome these problems, the existing protocols employ wake up schedules. Adaptive scheduling is employed in several of the MAC protocols. However, emergency traffic is random and unpredictable in nature. The delay is a major concern in the case of an emergency and may result in unwanted consequences if not handled promptly. Our aim is to solve this problem by using an external wake up mechanism. An out-of-band wake up mechanism can be used to avoid the idle waiting time of the nodes. The authors in [11,12] have proposed an out-of-band wake up radio system for sensor networks. The wake up radio mechanism works on the basic idea that a node can be woken up by an external trigger. It can be effectively used in an unscheduled environment.

In this paper, we propose a mechanism for emergency communication using a wake up radio. A node, when sensing an emergency event, can use the wake up radio to wake up the receiver node. Our aim is to prompt communication between nodes. Reducing the energy consumption and thereby increasing the lifetime are major design goals. The rest of the paper is organized as follows. In Section 2, we present related works and the motivation behind this work. In Section 3, we present the system design and modeling. In Section 4, we present the performance analysis. In Section 5, we present the performance evaluation. In Section 6, we present the sensitivity analysis. Finally, conclusions are drawn in Section 7.

## 2. Related Works and Motivation

The major applications of a BAN are in the healthcare domain. This is a very sensitive area. Therefore, the MAC protocols proposed for a BAN require extra care. The design of a MAC protocol for a BAN is influenced by several attributes [13]. Energy-efficient design incorporating low delay has been proposed by several authors in recent times. Some of these works focus on designing a comprehensive MAC protocol, while others focus on a particular application area (e.g., emergency handling). A wider range of MAC protocols is proposed. It has been observed that the time division multiple access (TDMA) and carrier sense multiple access (CSMA) mechanisms are the most popular in MAC protocols for a BAN. TDMA is preferred in earlier times, as it offers better performance in unsaturated traffic condition. The authors in [14] have proposed a TDMA-based MAC protocol design for a BAN. They have used the MAC frame structure with the uplink and downlink subframe using an adapting scheme. A simulation study with 24 nodes is presented. Their method tries to improve energy consumption using a pure TDMA scheme and does not use a contention-based mechanism. In the unscheduled wake up scheme, all of the nodes in the network have an independent wake up schedule. Since they do not know the wake up schedule of other devices, carrier sensing (CS) is employed to avoid collisions. WiseMAC, proposed in [9], is an example of such a MAC protocol. The collision-free time slot allocation method is proposed in [15]. The authors have proposed a distributed queuing body area network (DQBAN) MAC protocol using the process of the demand and deny time slot allocation scheme. They have used a cross-layer fuzzy-rule scheduling algorithm, which makes a node send a packet in the next frame instead of the first frame, thereby achieving better reliability. The evaluation shows a better packet success rate. MAC protocols using a hybrid wake up mechanism try to take advantage of both scheduled and unscheduled wake ups. An example of a hybrid wake up MAC is the IEEE802.15.4 MAC [16]. It supports the beacon-enabled and non-beacon-enabled modes of operation and uses contention and contention-free access mechanisms. Among the recent works on hybrid MAC, the authors in [17] proposed a cross-layer data dissemination approach for a BAN. It uses an adaptive priority scheme to reduce idle listening by the nodes and ensures effective traffic differentiation. The implementation shows lower energy consumption.

Emergency events are an integral part of a BAN. The authors in [18] have proposed a mechanism for emergency data transfer in a medical implant communication service (MICS)-based body area network. The authors in [19] have proposed an algorithm for priority-based allocation of time-slots (PATS) that considers a fitness parameter characterizing the criticality of health data that a packet carries, as well as the energy consumption rate for transmitting local data processing units. The authors in [20] have proposed the medical emergency body (MEB) MAC protocol that dynamically inserts listening windows and utilizes idle time slots to insert additional listening window opportunities for emergency traffic. The authors in [21] proposed a MAC protocol using an interrupt mechanism for medical monitoring applications. It is a hybrid MAC protocol and uses scheduled and contention access for data communication.

As mentioned earlier, the popular MAC protocols proposed for sensor networks can also be used in a BAN with trade-offs between energy consumption, lifetime and delay. Some of these protocols are S-MAC, T-MAC, Wise-MAC, B-MAC and X-MAC. For example, S-MAC, which uses a periodic sleep/wake up mechanism to conserve energy, can be used in a synchronized BAN. T-MAC has adaptive active time and can be used under variable traffic conditions. B-MAC uses a long preamble for communication. It can be used in a low traffic environment, as it has low energy consumption for low traffic and no synchronization overhead. The preamble sampling of WiseMAC is a strong candidate for BAN-related applications. It has low energy consumption and can adapt under variable traffic conditions. X-MAC, unlike B-MAC, uses small strobe preambles. It can be used for low delay applications. It also does not require synchronization, thereby reducing overheads. The IEEE 802.15.4 standard is devised to support low data rate, low power networks. The physical layer has 27 communication channels for industrial, scientific and medical (ISM) bands. The IEEE802.15.4 MAC has been proposed for a BAN [22]. It adopts carrier sense multiple access with collision avoidance (CSMA/CA) as the channel access mechanism. It supports a star topology and can be implemented in a typical BAN. The IEEE802.15.6 MAC [23] is a new standard protocol proposed for a BAN. It addresses both medical/healthcare and non-medical applications with diverse requirements. The MAC layer in the standard defines a short-range, wireless communication in and around the human body.

However, these protocols have several drawbacks. For example, the S-MAC protocol has a fixed periodic sleep and wake up mechanism. To reduce collisions among contending nodes, it uses the request to send (RTS) and clear to send (CTS) control packet’s handshake mechanism. However, this increases the overhead energy consumption. It also spends much time in idle listening, which causes energy waste. T-MAC improves the performance of S-MAC. However, it has an early sleeping problem. The receiver node waits for a fixed period of time, and after that, it goes to the sleep state if no activity is detected. At the same time, the idle listening period introduces overhead energy consumption. WiseMAC uses preamble sampling. It has low energy consumption and better performance under variable traffic conditions. The clock drift handling mitigates the external time synchronization requirement. The neighbor nodes may have different sleep and wake up times, which result in redundant transmission in the case of packet broadcast. This leads to higher energy consumption and latency. The probability of collision is high at the beginning of transmission of the preamble. The long preamble of B-MAC causes a longer delay. X-MAC is able to reduce the cost of a long preamble by using short strobe preambles. On average, it uses two preambles for one successful data transmission, which causes extra energy consumption. The approach used in these protocols is also known as the internal wake up mechanism due to the fact that these protocols use an internal clock to self-trigger a wake up. Another method is called external wake up, also known as the out-of-band wake up. In the next section, we present a detailed discussion of the external wake up mechanism.

### 2.1. Wake up Radio

An early work on an external radio-triggered wake up mechanism was proposed by the authors in [5] for sensor networks. They used a secondary transceiver called a wake up transceiver in each device in the network. It is controlled by a wake up circuit. A wake up receiver can respond immediately to the requests from other nodes, and the latency can be reduced.

The power consumption of the recently-developed wake up circuits is very small. These are in the range of a few μW compared to a few mW for the main radios used for data communication. The power consumption of some of the wake up radios is presented in Table 1.

For example, a basic wake up receiver presented in [11] needs only 84 μW for reception, whereas the CC1100 radio receiver consumes 29 mW. In other words, the wake up radio consumes only 0.03% of power compared to the CC1100 radio receiver. This in the long term contributes significantly to the conservation of power.

**Figure 2:**
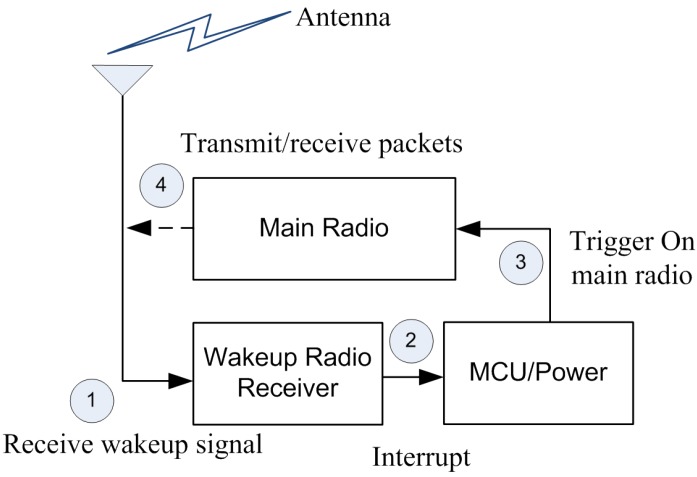
Wake up radio communication.

The goal of the wake up radio is to send a remote interrupt signal to a device. The basic working of a wake up radio includes sending an RF signal to a device in order to wake it up. The wake up radio system is powered by the energy received from the wake up radio signal. This energy is used to power up the components. After receiving the wake up signal, the target device powers on its main radio, as shown in Figure 2. An active wake up radio system can be used to send an acknowledgment back, confirming the reception of the wake up packet.

**Table 1:** Wake up radio and power consumption.

Wakeup Radio Model	Wakeup Power
Basic receiver [11]	84 μW
WuP [24]	470 nW
CargoNet [25]	4.8 μW
Ultra-Low Wakeup [26]	7.5 μW
Wakeup receiver [27]	171 μW
RTWAC [28]	2.6 μW
Picoradio [29]	1 μW

A wake up radio system saves energy by eliminating the causes of energy waste, such as idle listening, control packet overheads, overhearing and over-emitting. An external wake up radio system does not use specified wake up periods to listen to the channel for possible messages. In traditional MAC systems, a node has to turn on its main transceiver to listen to a channel. This uses much energy. However, a wake up radio system does not need idle listening and wake up periods. A wake up radio only responds to the low power incoming signals. It can keep its main radio in the off state and save energy. As explained in the next section, using addressing, it can also reduce the waking up of neighboring nodes.

The majority of the MAC protocols use control packets to establish the connection in the form of a handshake (request and permission for packet transmission) between two nodes. Beacon and poll packets are used for resource allocation. These packets are transmitted using the main radio. A wake up radio also uses handshake packets. However, the difference in energy consumption by the main radio and a wake up radio is large enough to affect the performance. Thus, the power that was previously dedicated to transmitting other control packets on the transmitter side and periodic monitoring on the receiver side can be replaced by the power consumption of the wake up radio system.

Another major problem is overhearing. The use of the main radio to overhear a possible packet transmission is very costly in terms of energy consumption. The popular CC2420 radio uses 35 mW for transmission and 38 mW for the reception. The CC1000 radio uses 42 mW for transmission and 29 mW for the reception. A wake up radio can complete the same task at a fraction of the energy required by the main radio, as is evident from Table 1. The energy use is in μW for the wake up radio and several mW in the case of the main radio. An external wake up radio helps to restrict the use of the main radio for data packet transmission only, thereby saving a significant amount of energy.

The use of a wake up radio-based system can benefit a BAN, as it can save a significant amount of energy. This can in turn help increase the network lifetime. In our work, we propose to use an ultra low power wake up signal to trigger the wake up circuit inbuilt in a BAN node.

#### 2.1.1. Wake up Radio Packet and Addressing

**Figure 3:**
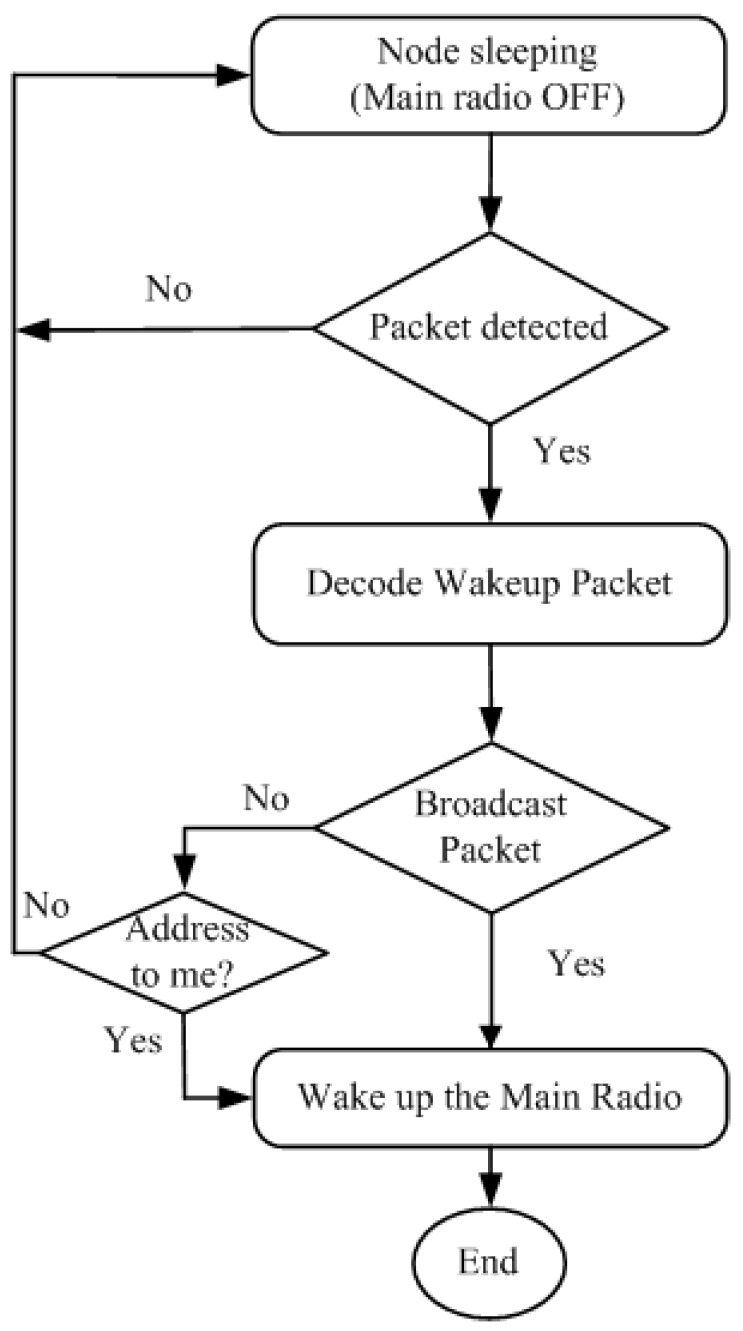
Flow of the wake up radio system with addressing.

Addressing is an important factor in the wake up radio. It is used for selective communication. A flow chart of a typical wake up radio-based system using addressing is shown in Figure 3. It is to be noted that energy is consumed to decode a wake up packet to determine the recipient. However, unlike the originally-proposed wake up radio models [5,11], addressing can reduce the waking up of all of the nodes in the neighborhood with a slight increase in the complexity [30].

#### 2.1.2. Implementation of the Wake up Radio

**Figure 4:**
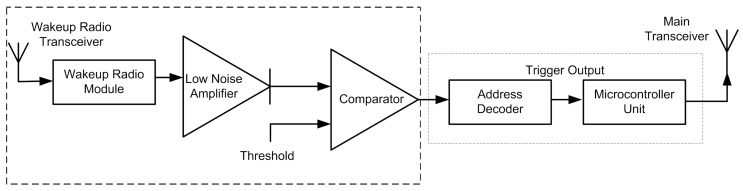
Example of the wake up radio architecture.

A wake up radio can be implemented using simple hardware, as shown in Figure 4. A working implementation of a wireless sensor node with a wake up radio is presented in [27]. It consists of a wake up radio circuit attached to the T-node platform. It consists of an 8-MHz ATmega128L (Atmel Corporation, San Jose, CA, USA)) processor and Chipcon CC1000 radio, which operates in the European license-free 868-MHz band. It uses on-off keying (OOK) modulation with a symbol rate of 862 Hz.

**Figure 5:**
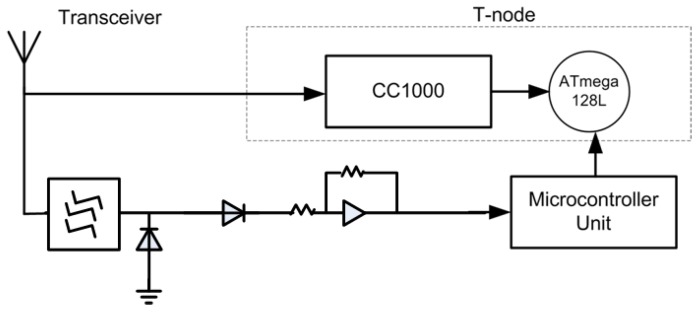
A wake up radio attached to the T-node.

The high-level design of the wake up circuitry and its interface to the T-node components is shown in Figure 5 [27]. At first, the incoming signals from the antenna are fed through a frequency filter to suppress external interference (mainly GSM signals at the 900-MHz band). Then, the 868-MHz signal is converted down to a low-frequency baseband signal using a simple diode. It is followed by a large amplification to make the signal detectable by an ultra-low-power microcontroller. This microcontroller applies some digital processing to filter the residual (self) interference still present in the input signal. Once the wake up signal is detected, the ATmega128L processor of the T-node is triggered into action by means of an interrupt, which will in turn switch on the CC1000 radio to receive the data packets [27].

**Figure 6:**
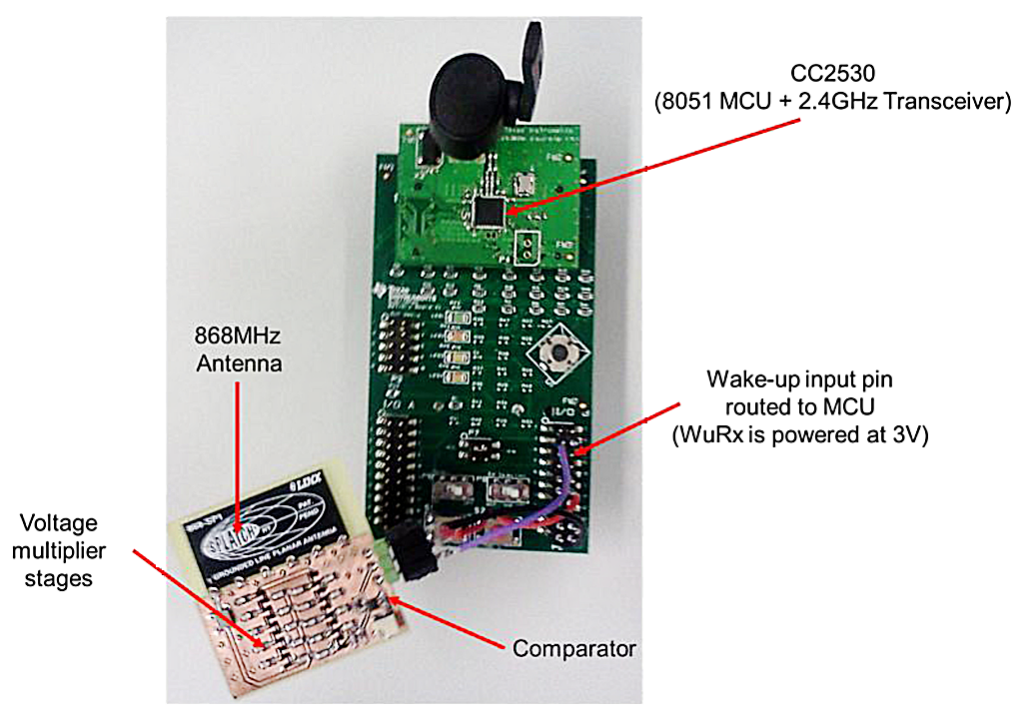
A low-complexity wake up receiver (WuRx) attached to a wireless sensor node.

A wake up signal is transmitted at 3 mW from 10 m away and attenuated to a level of −51 dBm, which is equivalent to 10 μV peak-to-peak on the antenna line. After an attenuation of 3.5 dB caused by the frequency filter, this reduces to a mere 5 μV. It is used with a CC2530 chip using the 2.4-GHz transceiver. In another work, the authors in [31] have implemented a system for sensor networks using a low complexity wake up radio, as shown in Figure 6. The clock rate of the programmable intelligent computer (PIC) can be either increased to the next level at 125 kHz, or the frequency of the wake up signal can be lowered. It is to be noted that the PIC also contains an internal comparator, but operating it consumes at least 90 μW due to the need to keep it on continuously because of its longer setup times.

PicoRadio [29] is one of the earliest works on wake up radio monitoring in the environment. It uses a very low power transceiver module. RTWAC [28] is a radio triggered wake up with addressing capability that allows suppressing current consumption during the idle state. It consists of an external low-cost hardware wake up circuit attached to the microcontroller of the sensor device. In [29], the authors have proposed a radio-triggered wake up circuit to control the operation of sensor devices. The authors in [32] have proposed a dual-radio cooperation that uses a low-power wake up radio to minimize the energy consumption of the wireless node in low event rates. All of these works show that the wake up radio is a promising technology. It can be effectively used for short-range communication.

### 2.2. Motivation behind Using the Wake up Radio for BAN

A BAN can use the wake up radio to reduce the energy consumption and prolong the lifetime of the nodes. It can be used for both emergency and regular (normal) communication. For regular communication, a major motivation is that a node should wake up only when there is a need for communication, or else, it should remain in the sleep state and conserve energy. A wake up radio transceiver added to each node can help to meet this goal. Unnecessary wake up periods for a node can be avoided, which can minimize energy consumption. This in turn increases the lifetime of the nodes. There is a lack of a satisfactory means to communicate immediately in current protocols, and delay is a major issue. This is also true in the case of the IEEE15.x standard protocols.

#### 2.2.1. Sensitivity of the Wake up Radio

The sensitivity of a wake up radio receiver can be derived using the Friis free space equation.

(1) $$P_{r}=\frac{P_{t}G_{t}G_{r}}{{\left(4\pi \right)}^{2}}{\left(\frac{L}{d}\right)}^{n}$$

where *L* is the wavelength of the signal, *d* is the distance, *n* is the empirical path loss exponent, $P_{t}$ is the transmitted power, $G_{t}$ is the antenna gain for the transmitter and $G_{r}$ is the antenna gain for the receiver. We can find a reasonable range for the wake up receiver with acceptable sensitivity. As mentioned earlier, in an application like a BAN, a short range of 3–5 m is adequate. The traditional 2.4-GHz ISM band can be used for the wake up signal.

If we assume $G_{t}$ = $G_{r}$ = 1.5 dBi and a radiated power Pt = 0 dBm, we can find the received power (in dBm). It is given by,

(2) $$P_{r}=-37.07-10n\log d$$

It can be seen from Table 2 that we can use a wake up radio with sensitivity −58 dBm in a BAN for a range up to 5 m. The sensitivity can be increased with higher antenna gains. The power usage limits the range of a typical wake up radio. However, this makes itself suitable for short-range applications, such as a BAN.

**Table 2:** Sensitivity of the wake up radio.

Distance (m)	*n*	Pr (dBm)
3	2	−46.61
	3	−51.38
5	2	−51.05
	3	−58.04

**Figure 7:**
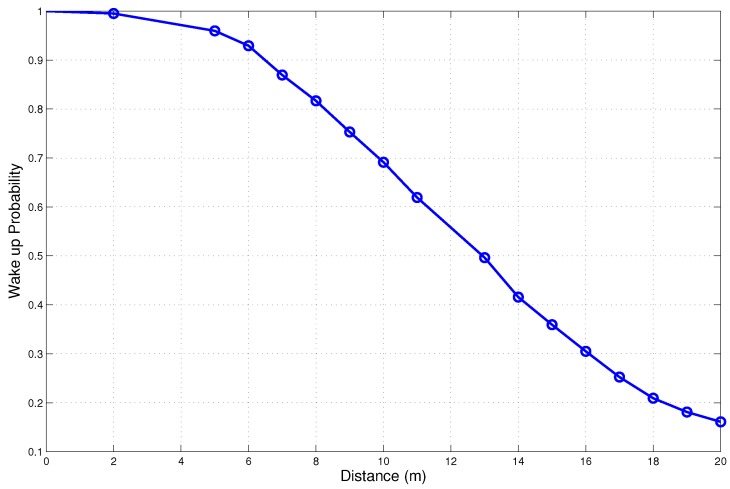
Wake up probability *vs.* distance.

A wake up radio-based system through an on-demand request can significantly reduce the idle state energy consumption. A typical BAN has a 3–5 m coverage area. As seen in Figure 7, a BAN can take advantage of the high wake up probability at short range [33]. In addition, there is only a very limited impact on latency because the corresponding node wakes up immediately. In recent times, wake up radios intended for a BAN have been researched. Several works are underway to further improve the receive power. The authors in [24] developed a nano-power wake up radio for a BAN.

The wake up radio-based MAC that we propose here takes advantage of a typical BAN network as follows:

- a smaller network size in terms of nodes compared to typical WSN;
- limited communication range;
- a BAN node can be easily triggered by an external wake up radio signal;
- the wake up radio requires little extra cost in terms of power consumption.

## 3. System Design and Modeling

In this section, we describe the design of the proposed system.

### 3.1. Emergency Traffic Communication

Emergency events in a BAN can occur due to several reasons. It may happen in any of the BNs, including the BNC. For example, a BN can sense abnormality in the sensing data or it can sense that the battery is dying. The BNC may face critical problems during its operation. It may also require immediate data from a node that is currently in the sleep state. All of these can be classified as an emergency or urgent task. The tasks can be medical health related or non-medical in nature. The handling of the emergency event is a very sensitive issue in a BAN. This is particularly true for an emergency occurring for the implant nodes. The delay must be as low as possible to handle such situations.

### 3.2. Communication Process

**Figure 8:**
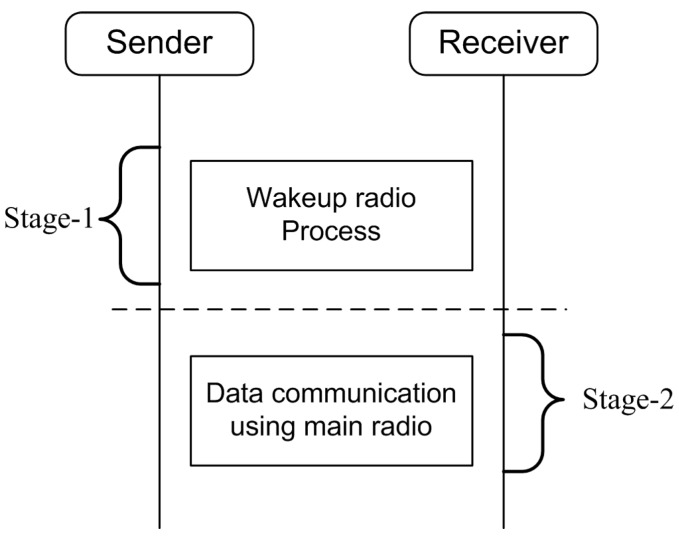
Communication process.

A wake up process is handled using the wake up radio. A two-stage communication process is used as shown in Figure 8. In Stage 1, the wake up radio is switched on. Once the receiver node verifies itself as the intended receiver, it transmits back an acknowledgment to the sender using the same channel. In Stage 2, the main radio transceivers are triggered on for data communication if required.

**Figure 9:**
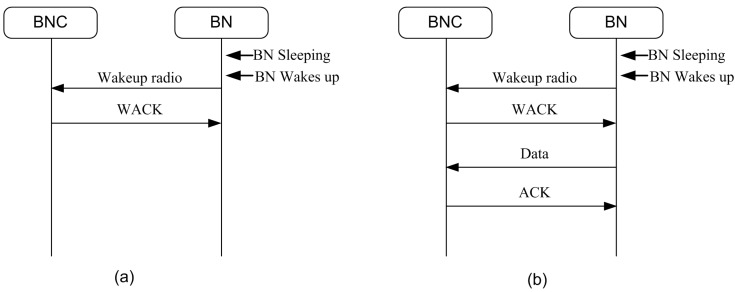
Emergency communication: (**a**) without data; (**b**) with data.

An example of the emergency communication process is shown in Figure 9. In the first case, we have depicted the case of an emergency wake up command (emergency alarm) packet. This process is completed in Stage 1 itself. It can be used to notify about emergency types, which the receiver can know by looking into predefined information in the wake up packet. The emergency command can be described as a short wake up frame (SWUF). The sender then waits for the wake up acknowledgment (WACK) timeout period. It retransmits the command if no WACK is received. The process continues until successful. The second case depicts an on-demand data communication process. This can be used for both emergency and normal (regular) data communication. In this case, the wake up process is followed by the data communication process.

**Figure 10:**
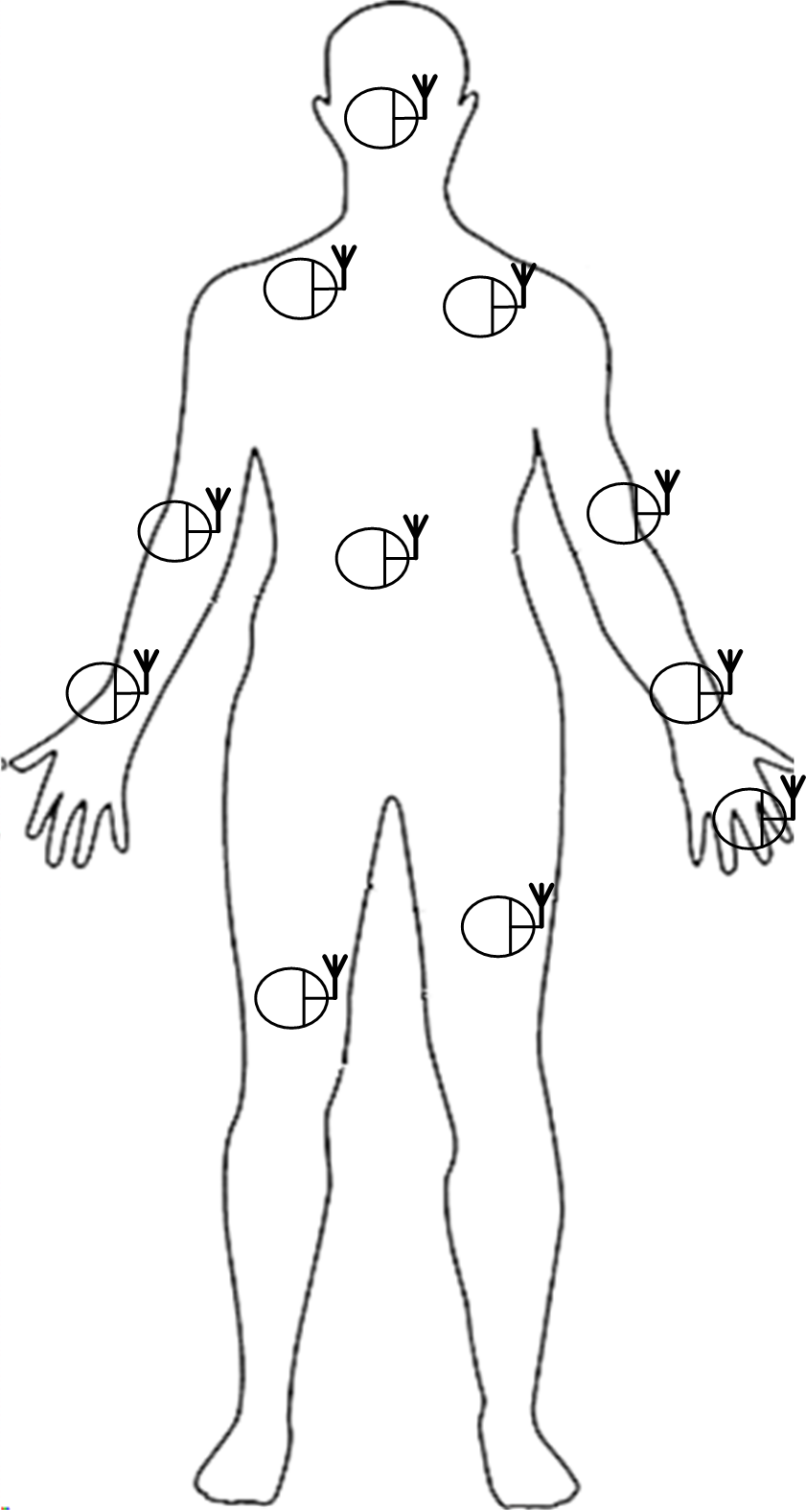
BAN network setup.

All of the BNs in the network are assumed to be equipped with an antennae for the wake up radio and data communication. A typical configuration is shown in Figure 10. The number of BNs and their positions are changed during the evaluation. A BN is capable of both receiving and sending the wake up radio signal. A BN remains in the sleep state until either an event triggers it on or it is woken up by an external radio signal.

### 3.3. Network Setup

**Figure 11:**
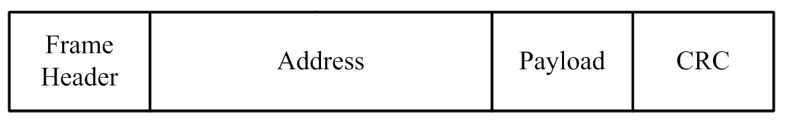
Wake up packet.

**Figure 12:**
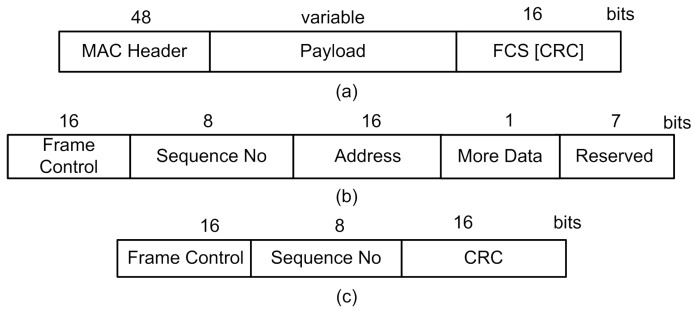
Frames: (**a**) MAC; (**b**) header; (**c**) ACK.

A typical wake up packet uses the address of a node, as shown in Figure 11. The fields in the wake up packets are: frame header, address, payload and frame check sequence (FCS) using the cyclic redundancy code (CRC) algorithm. The frame header contains a preamble and start frame delimiter (SFD). They help against miss and false detection and provide synchronization. The node address or ID is used to identify the intended receiver. The payload contains information about the events. Other MAC frames used for simulation purposes are shown in Figure 12. We have used a “more data” field for multiple packets transmission. One bit is used to depict a simple yes/no for more data packets. The final packet size depends on the payload field. The physical (PHY) layer packet properties are borrowed from the IEEE802.15.4 channel model.

## 4. Performance Analysis

**Figure 13:**
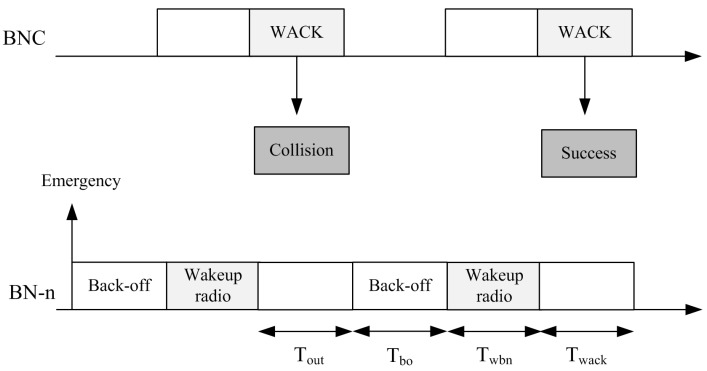
Example MAC operation using the wake up radio.

A slotted contention-based mechanism is used for communication. An example MAC operation is shown in Figure 13. A BN with an emergency event uses channel sensing to check the channel for activity. It also uses the back-off mechanism to avoid collisions. In the next section, we discuss the back-off mechanism.

### 4.1. Back-Off Mechanism

**Figure 14:**
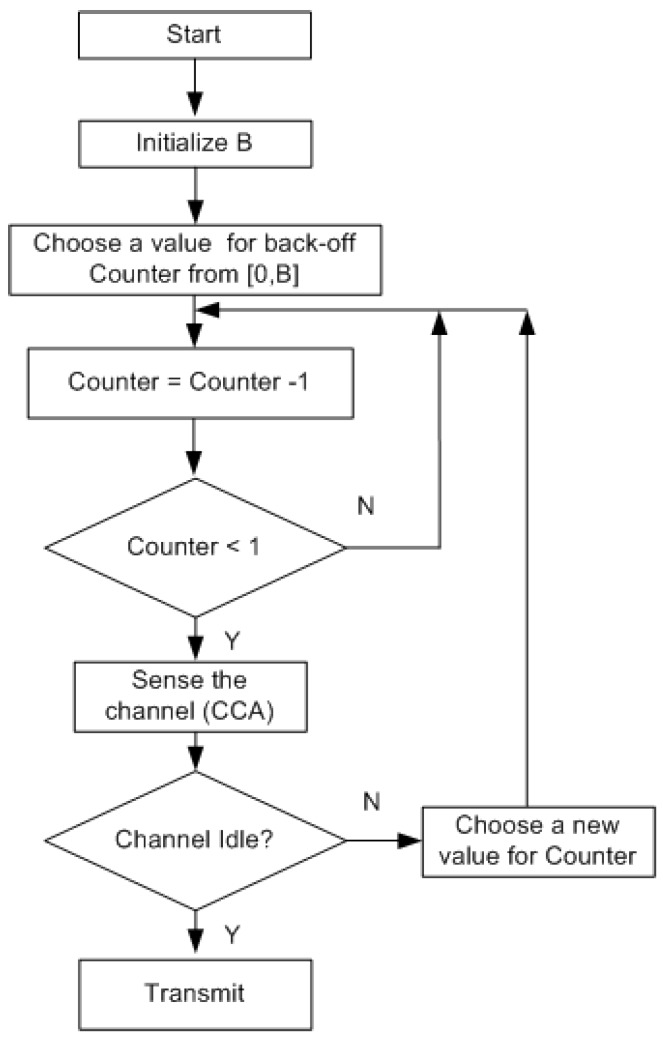
Back-off mechanism.

Before attempting to transmit, a BN utilizes the back-off mechanism. It chooses the value from the range [0, B], where the back-off window size (B) can be fixed or adapted as per the requirements. The value a BN chooses is called the back-off counter. It is expressed in terms of slots. The counter value is decremented one slot at a time. For example, if a BN chooses a back-off value of three, it waits for three slots before reattempting to transmit the packet. Once the counter expires, the BN senses the channel. If the channel is idle, it transmits the wake up radio packet. If it senses the is channel busy, it chooses a new value for the counter, and the process is repeated. It is to be noted that a number of back-off algorithms can be used depending on their feasibility in a particular BAN application. The flow chart of the back-off mechanism is shown in Figure 14.

The average back-off size ($\bar{B}$) is given by,

(3) $$\bar{B}=\frac{B-1}{2}$$

In this work, we have assumed a Poisson distribution for traffic. For a network with N nodes, the total rate is given by Nα. For analysis, we consider the two periods, idle and active. The idle period is the time when a BN does not transmit or receive. In the idle period, a BN can shut down the transceivers and go to the sleep state. Thus, a BN is said to be idle from the end of the last packet transmission until the start of the next transmission. Similarly, a BN is active from the moment a transmission starts until the end of the transmission. The notations used in this work are presented in Table 3.

**Table 3:** Description of notations.

Notation	Description	Units
N	Number of nodes	-
$T_{s}$	Slot duration	ms
$T_{x}$	Duration between packet arrivals	ms
$T_{i}$	Idle period	ms
$T_{a}$	Active period	ms
$T_{wk}$	Wake up radio duration	ms
$T_{wbn}$	Wake up packet transmission time	ms
$T_{wack}$	Wake up acknowledgment time	ms
*λ*	Arrival rate	packet/s
*α*	Attempt rate	packet/s
A	Random time between the first and last packets	ms
$p_{b}$	Busy channel probability	-
$T_{at}$	Mean busy period to transmit the wake up packet	ms
$T_{ar}$	Mean busy period to receive the wake up ACK	ms
$T_{tx}$	Time a BN spends in transmitting	ms
$T_{rx}$	Time a BN spends in receiving	ms
$T_{id}$	Time a BN spends in idle state	ms
E	Energy consumption by a BN	mW
$E_{ov}$	Overhead energy consumption	mW
$T_{wd}$	Waiting time when channel is initially idle	ms
$T_{ed}$	Average time for the initial successful attempt	ms
B	Back-off window size	slots
D	Delay	ms
$p_{s}$	Probability of successfully transmitting the packet	-
$T_{life}$	Lifetime	days
CCA	Clear channel assessment	ms
$P_{tx}$	Transmitting power	mW
$P_{rx}$	Receiving power	mW
$L_{wack}$	Length of wake up acknowledgment packet	bytes
$T_{sw}$	Transceiver switching time	ms
$P_{sw}$	Transceiver switching power	mW
$T_{tr}$	Transition time	ms
$P_{tr}$	Transition power	mW
$T_{cs}$	Time required for CCA	ms
$P_{cs}$	Power required for CCA	mW
r	Data rate	kbps

### 4.2. Energy Consumption

The cumulative distribution of the duration from the end of the last packet transmission until the start of the arrival of the next packet ($T_{x}$) is given by,

(4) $$P\left(T_{x}\leq x\right)=1-P\left(T_{x}>x\right)=1-e^{-N\alpha x}$$

The mean is given by,

(5) $$E\left[T_{x}\right]=\frac{1}{N\alpha }$$

This is equal to the mean duration of the idle period.

Therefore,

(6) $$E\left[T_{i}\right]=E\left[T_{x}\right]=\frac{1}{N\alpha }$$

The active period is given by,

(7) $$T_{a}=T_{wk}+A$$

where $T_{wk}$ is the summation of the time required to send and receive the wake up radio packets and is given by,

(8) $$T_{wk}=T_{wbn}+T_{wack}$$

The parameter A is a random variable with a cumulative distribution as follows,

(9) $$P\left(A\leq a\right)=1-e^{-\left(N-1\right)\alpha \left(T_{s}-a\right)}$$

The probability distribution function is given by,

(10) $$\begin{array}{cc} f_{A}\left(a\right) & =\frac{d}{da}P\left(A\leq a\right)\\ & =e^{-\left(N-1\right)\alpha T_{s}\partial \left(a\right)}+\left(N-1\right)\alpha e^{-\left(N-1\right)\alpha \left(T_{s}-a\right)} \end{array}$$

In this case, the mean is given by,

(11) $$E\left[A\right]=T_{s}-\frac{1-e^{-\left(N-1\right)\alpha T_{s}}}{\left(N-1\right)\alpha }$$

Therefore, the mean active period is given by,

(12) $$E\left[T_{a}\right]=T_{wk}+T_{s}-\frac{1-e^{-\left(N-1\right)\alpha T_{s}}}{\left(N-1\right)\alpha }$$

We calculate the energy consumption considering the successful transmission during an event. The SWUF is used to inform the receiver about the emergency event. Let $p_{b}$ be the busy channel probability during the active period. The corresponding idle channel probability is given by ($1-p_{b}$). During an active period, we assumed that the transmission follows the Poisson process. In this case, the rate is given by $\alpha (1-p_{b})$.

In an active period at the BN, it spends some time in the idle period. The cumulative distribution of this duration is given by,

(13) $$\begin{array}{cc} P\left(T_{ai}\leq x\right) & =1-P\left(T_{ai}>x\right)\\ & =1-e^{-\left(1-p_{b}\right)\alpha x} \end{array}$$

The mean in this is given by,

(14) $$E\left[T_{ai}\right]=\frac{1}{\left(1-p_{b}\right)\alpha }$$

The mean busy period for transmitting the wake up packet is given by,

(15) $$E\left[T_{at}\right]=T_{wbn}$$

The mean busy period for receiving the wake up acknowledgment packet is given by,

(16) $$E\left[T_{ar}\right]=T_{wack}$$

Now, we find the transmitting and receiving time for the BN. The proportion of time a BN spends in the transmitting state is given by,

(17) $$T_{tx}=\frac{E\left[T_{at}\right]}{E\left[T_{ai}\right]+E\left[T_{at}\right]+E\left[T_{ar}\right]}$$

The proportion of time a BN spends in the receiving state is given by,

(18) $$T_{rx}=\frac{E\left[T_{ar}\right]}{E\left[T_{ai}\right]+E\left[T_{at}\right]+E\left[T_{ar}\right]}$$

We can find the expression for $T_{tx}$ and $T_{rx}$ as follows,

(19) $$\begin{array}{cc} T_{tx} & =\frac{T_{wbn}}{\frac{1}{\left(1-p_{b}\right)\alpha }+T_{wbn}+T_{wack}}\\ & =\frac{\left(1-p_{b}\right)\alpha T_{wbn}}{1+\left(1-p_{b}\right)\alpha T_{wbn}+\left(1-p_{b}\right)\alpha T_{wack}} \end{array}$$

(20) $$\begin{array}{cc} T_{rx} & =\frac{T_{wack}}{\frac{1}{\left(1-p_{b}\right)\alpha }+T_{wbn}+T_{wack}}\\ & =\frac{\left(1-p_{b}\right)\alpha T_{wack}}{1+\left(1-p_{b}\right)\alpha T_{wbn}+\left(1-p_{b}\right)\alpha T_{wack}} \end{array}$$

The proportion of time a BN spends in the idle state is given by,

(21) $$T_{id}=\frac{E\left[T_{a}\right]}{E\left[T_{i}\right]+E\left[T_{a}\right]}-T_{tx}-T_{rx}$$

Thus,

(22) $$\begin{array}{c} T_{id}=\frac{T_{wk}+T_{s}-\frac{1-e^{-\left(N-1\right)\alpha T_{s}}}{\left(N-1\right)\alpha }}{\frac{1}{N\alpha }+T_{wk}+T_{s}-\frac{1-e^{-\left(N-1\right)\alpha T_{s}}}{\left(N-1\right)\alpha }}-\frac{\left(1-p_{b}\right)\alpha T_{wbn}}{1+\left(1-p_{b}\right)\alpha T_{wbn}+\left(1-p_{b}\right)\alpha T_{wack}}-\frac{\left(1-p_{b}\right)\alpha T_{wack}}{1+\left(1-p_{b}\right)\alpha T_{wbn}+\left(1-p_{b}\right)\alpha T_{wack}} \end{array}$$

The energy consumption (*E*) by the BN is calculated by adding the energy consumption in all of the states, transmitting, receiving and idle, including the overheads, and is given by,

(23) $$E=T_{tx}P_{tx}+T_{rx}P_{rx}+T_{id}P_{id}+E_{ov}$$

where $E_{ov}$ is the overhead energy consumption. It is given by,

(24) $$E_{ov}=P_{tr}T_{tr}+P_{sw}T_{sw}+P_{cs}T_{cs}$$

### 4.3. Delay

The delay is calculated starting from the time of channel sensing till the acknowledgment is received. As mentioned earlier, when the BN detects an event, it senses the channel. If it initially finds the channel idle, it transmits the packet. However, in the case of the busy channel, it invokes the back-off process and waits till the back-off counter expires.

If the BN initially finds the channel idle, it transmits the packet. In this case, the average waiting time is given by,

(25) Twd=B¯B¯1-pb1-pb

The parameter $p_{b}$ is given by,

(26) $$p_{b}=\frac{E\left[T_{a}\right]}{E\left[T_{i}\right]+E\left[T_{a}\right]}$$

Then, the average waiting time is given by,

(27) Twd=B¯B¯1-Twk+Ts-1-e-N-1αTsN-1α1Nα+Twk+Ts-1-e-N-1αTsN-1α1-Twk+Ts-1-e-N-1αTsN-1α1Nα+Twk+Ts-1-e-N-1αTsN-1α

The average time for the initial successful attempt is given by,

(28) $$T_{ed}=T_{wd}+T_{wk}$$

Thus, we have,

(29) Ted=B¯B¯1-Twk+Ts-1-e-N-1αTsN-1α1Nα+Twk+Ts-1-e-N-1αTsN-1α1-Twk+Ts-1-e-N-1αTsN-1α1Nα+Twk+Ts-1-e-N-1αTsN-1α+Twk

Now, we take into account the fact that a BN retransmits the wake up radio packet once the WACK time expires, *i.e.*, it does not receive the WACK from the receiver. During retransmission, the BN again invokes the back-off procedure.

The delay (*D*) until the wake up packet is successfully transmitted is given by,

(30) $$D=\frac{T_{ed}}{p_{s}}$$

The probability of successfully transmitting the wake up packet $p_{s}$ is given by,

(31) $$p_{s}=e^{-2\left(N-1\right)\alpha T_{s}}$$

Thus, we have,

(32) D=B¯B¯1-Twk+Ts-1-e-N-1αTsN-1α1Nα+Twk+Ts-1-e-N-1αTsN-1α1-Twk+Ts-1-e-N-1αTsN-1α1Nα+Twk+Ts-1-e-N-1αTsN-1α+TwkB¯B¯1-Twk+Ts-1-e-N-1αTsN-1α1Nα+Twk+Ts-1-e-N-1αTsN-1α1-Twk+Ts-1-e-N-1αTsN-1α1Nα+Twk+Ts-1-e-N-1αTsN-1α+Twke-2N-1αTse-2N-1αTs

### 4.4. Lifetime

The lifetime $T_{life}$ (in number of days) is calculated using the following expression,

(33) $$T_{life}=\frac{InitialBatteryEnergy}{E\times 365\times 24}$$

### 4.5. Analysis for Other Systems

We have compared our model against some of the well-known state-of-the-art MAC protocols. In this section, the expressions for energy consumption and delay are presented.

Let $E_{WiseMAC}$, $E_{XMAC}$, $E_{BMAC}$, $E_{TMAC}$, $E_{SMAC}$, $E_{154}$ and $E_{156}$ be the average energy consumption of the WiseMAC, X-MAC, B-MAC, T-MAC, S-MAC, IEEE802.15.4 and IEEE802.15.6 MAC protocols, respectively. The expressions for the average energy consumption are as follows:

(34) $$E_{WiseMAC}=\frac{P_{tx}\times \left(\frac{\frac{1}{R}}{T_{wk}}+\left(\bar{X}+T_{data}\right)\right)+P_{rx}\times \left(T_{ack}+\bar{Y}\times \left(N-1\right)+T_{px}\right)}{T}+E_{ov}$$

where:

(35) $$\bar{X}=2L\left(1-e^{-\frac{T_{data}}{4L}}\right)$$

(36) $$\bar{Y}=\frac{{T^{2}}_{data}+12LT_{data}}{2T_{wk}}\left(1-e^{-\frac{T_{wk}}{4L}}\right)$$

(37) $$E_{XMAC}=\frac{N_{probe}\times \left[\left(P_{tx}\times T_{p}\right)+\left(P_{rx}\times T_{ack}\right)\right]+P_{tx}\times T_{data}+P_{rx}\times \left(T_{ack}+\left(2T_{p}+T_{ack}\right)\times \left(N-1\right)+T_{px}\right)}{T}+E_{ov}$$

(38) $$E_{BMAC}=\frac{P_{tx}\times \left(T_{p}+T_{data}\right)+P_{rx}\times \left(T_{ack}+\left(\frac{T_{wk}}{2}-T_{cs}\right)+T_{px}\right)+T_{data}\times \left(N-1\right)}{T}+E_{ov}$$

(39) $$E_{TMAC}=\frac{P_{tx}\times \left(T_{rts}+T_{data}\right)+P_{rx}\times \left(T_{ack}+T_{cts}\times \left(N-1\right)+T_{px}\right)}{T}+E_{ov}$$

(40) $$E_{SMAC}=\frac{P_{tx}\times \left(T_{rts}+T_{data}\right)+P_{rx}\times \left(T_{ack}+T_{cts}\times \left(N-1\right)+T_{syn}\right)}{T}+E_{ov}$$

(41) $$E_{154}=\frac{P_{tx}\times T_{data}+P_{rx}\times \left(T_{ack}+4T_{wk}+2T_{b}\times \left(N-1\right)+T_{px}\right)}{T}+E_{ov}$$

(42) $$E_{156}=\frac{P_{tx}\times T_{data}+P_{rx}\times \left(T_{ack}+T_{wk}+T_{px}\right)}{T}+E_{ov}$$

Let $D_{WiseMAC}$, $D_{XMAC}$, $D_{BMAC}$, $D_{TMAC}$, $D_{SMAC}$, $D_{154}$ and $D_{156}$ be the delay of the WiseMAC, X-MAC, B-MAC, T-MAC, S-MAC, IEEE802.15.4 and IEEE802.15.6 MAC protocols, respectively. The expressions for the delay are as follows:

(43) $$D_{WiseMAC}=T_{tr}+T_{data}+T_{ack}+T_{sw}+T_{px}+\frac{T_{wk}}{2}$$

(44) $$D_{XMAC}=T_{tr}+T_{data}+2T_{ack}+N_{probe}\times T_{p}+3T_{sw}+T_{px}$$

(45) $$D_{BMAC}=T_{tr}+T_{data}+T_{ack}+T_{p}+T_{sw}+T_{px}$$

(46) $$D_{TMAC}=T_{tr}+T_{data}+T_{ack}+T_{sw}+T_{a}+T_{cs}$$

(47) $$D_{SMAC}=T_{tr}+T_{cs}+T_{data}+T_{ack}+T_{sw}+T_{rts}+T_{cts}+T_{syn}$$

(48) $$D_{154}=T_{tr}+2T_{cs}+T_{data}+T_{ack}+T_{sw}+T_{px}$$

(49) $$D_{156}=T_{tr}+T_{cs}+T_{data}+T_{ack}+T_{sw}+T_{px}$$

## 5. Performance Evaluation

In this section, we present a discussion of the analytical and simulation results of the proposed model.

### 5.1. Experimental Setup

**Figure 15:**
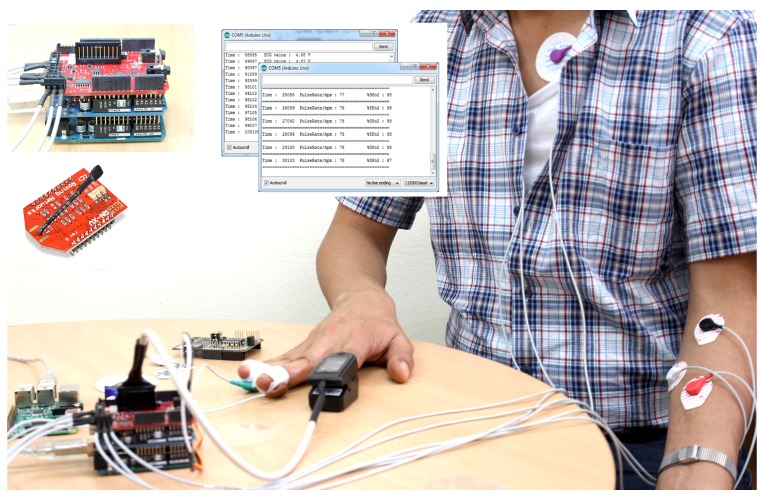
Experimental setup.

We set up an experiment using both wired and wireless sensor nodes, as shown in Figure 15. The experimental evaluation is done on the open source Arduino Health platform [34]. It supports biometric and medical applications where body monitoring is needed using sensors. It has a 16-MHz Atmega 328 processor with an operating voltage of 5 V, static random-access memory (SRAM) 2 kb, electrically erasable programmable read-only memory (EEPROM) 1 kb. It accepts digital and analog I/Os. It can seamlessly incorporate the eHealth and wireless modules. The sensor devices used in the experiment are as follows: electrocardiogram (EEG), muscle electromyography (EMG), body temperature, blood pressure (BP), galvanic skin response (GSR), pulse oximeter (SpO2), position and accelerometer sensors. The experiment helped to produce scheduled data for various devices for the normal traffic in the network. The inter-arrival time for normal traffic is tested in the network. The data are then used for the simulation.

### 5.2. Simulation Setup

**Figure 16:**
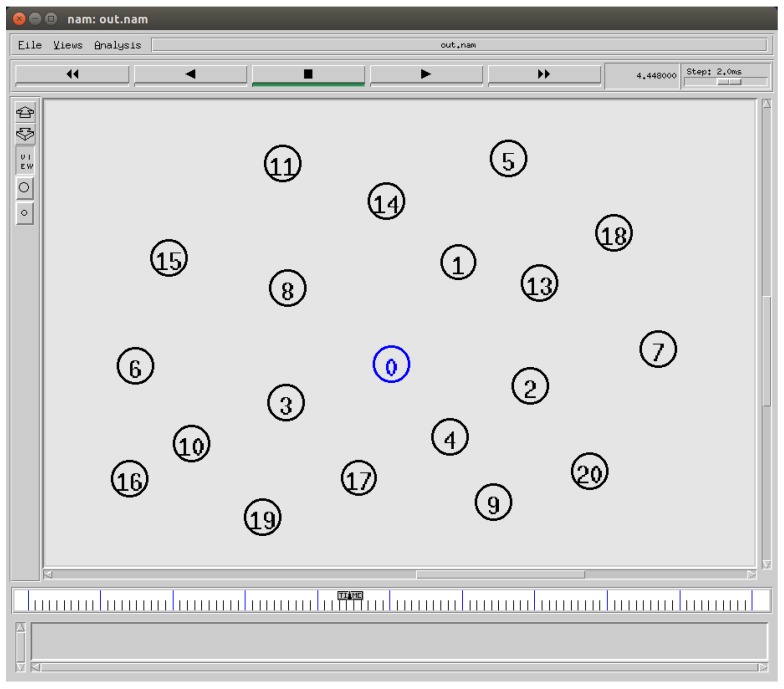
A simulation scenario generated in NS2.

The simulation is performed to validate the model. The BNs are deployed at a one-hop distance from the BNC. The position of each BN is randomized for the simulation. The average distance between the nodes varied from 0.1 m–5.0 m. Each BN is assumed to have a wake up radio transceiver. We set the simulation to transmit a wake up command. The occurrence of the emergency is varied from a small number of events to a large number of events. The number of BNs in the system is also varied. The energy consumption, delay and lifetime are calculated. We have taken the mean of 50 simulation runs. The nodes changed position in each simulation scenario. An example of a generated scenario is shown in Figure 16. Node 0 acts as the BNC. The traffic is asymmetrical. Both the BNC and BNs generate the traffic with a particular arrival rate.

The input parameters for the simulation work are presented in Table 4. We have used the open source Network Simulator NS-2 (release v2.35) tool for simulation work. The Tcl is used for scripting the network along with C++ codes. The rest of the MAC specific parameters as presented in Table 5 are taken from their respective works [6,7,8,9,10].

**Figure 17:**
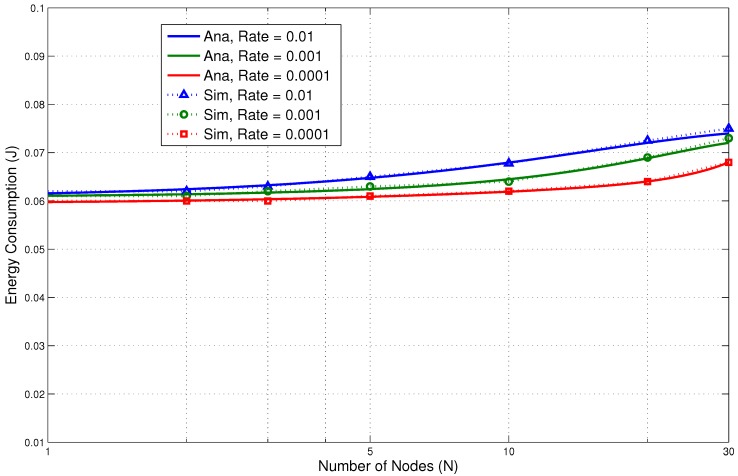
Emergency traffic energy consumption.

**Figure 18:**
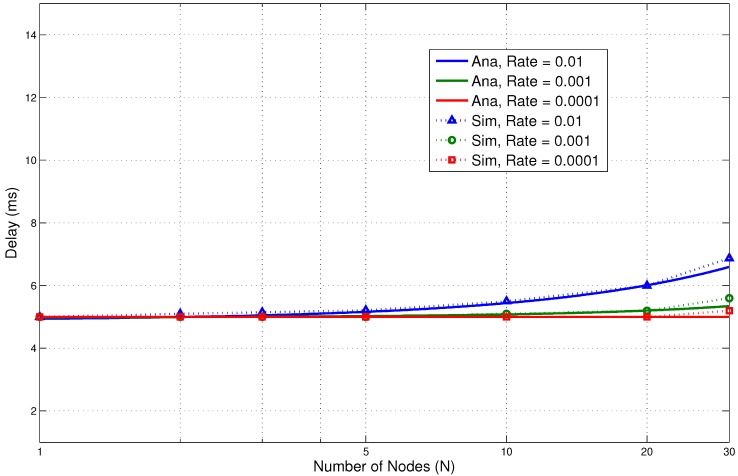
Emergency traffic delay.

**Figure 19:**
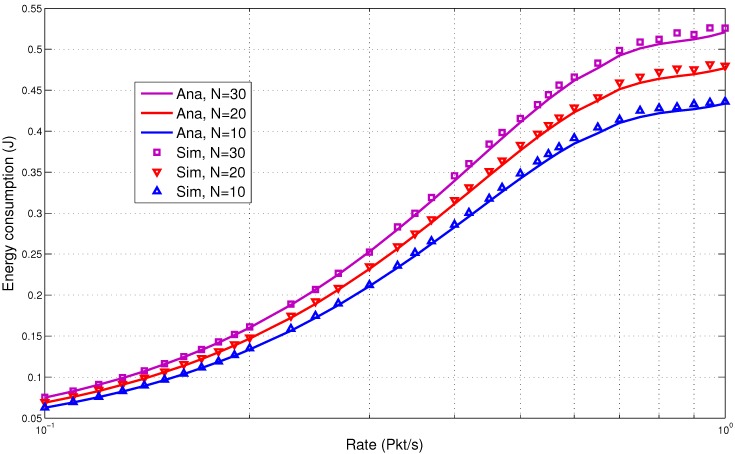
Combined traffic energy consumption.

**Figure 20:**
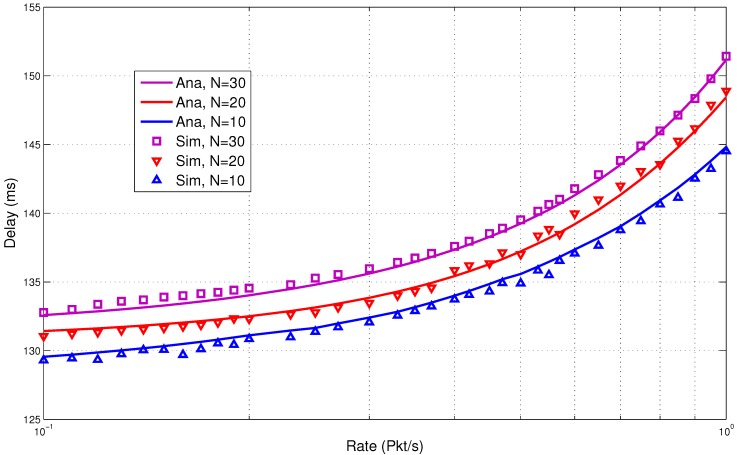
Combined traffic delay.

**Figure 21:**
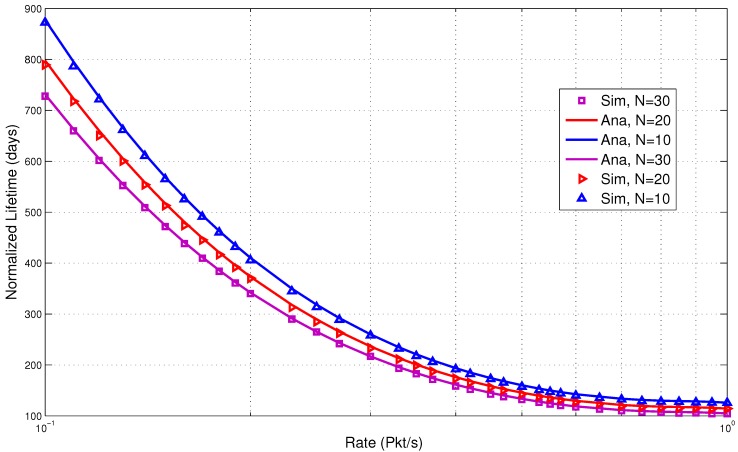
Combined traffic lifetime.

In the next section, we discuss the performance results. They clearly show low energy consumption with a lower delay. This is due to the fact that the design of the proposed protocol is able to reduce the packet overheads and idle listening, which are major causes of energy consumption. It is very important that these overheads are avoided to increase the overall performance of the system. Comparisons of the results of the analytical and simulation models are presented in Figure 17, Figure 18, Figure 19, Figure 20 and Figure 21. The legends “Ana” and “Sim” in the figures represent analytical and simulation results, respectively. The closeness in the results of the analysis and simulation validates our model.

**Table 4:** Input parameters. SWUF, short wake up frame.

Symbol	Value	Symbol	Value
$P_{tx}$	26 mW	$P_{rx}$	13.5 mW
$L_{wack}$	6 B	$P_{sw}$	13.5 mW
$T_{sw}$	0.4 ms	$P_{tr}$	0.004 mW
$T_{tr}$	0.25 ms	*λ*	Variable
r	25 kbps	B	32
Data	Variable	ACK	10 B
SWUF	8 B	N	Variable
$T_{s}$	7.68 ms	CCA	3 ms
$P_{wbn}$	1.4 mW	$P_{wack}$	0.084 mW

**Table 5:** MAC-specific input parameters. RTS, request to send; CTS, clear to send.

WiseMAC		X-MAC		B-MAC	
$T_{wk}$	400 ms	$T_{wk}$	43.35 ms	$T_{wk}$	400 ms
$T_{p}$	20 ms	$T_{p}$	2.41 ms	$T_{p}$	86.7 ms
θ	30 ppm	Probe	35.65 ms		
**T-MAC**		**S-MAC**		**IEEE802.15.4/6**	
Active time	15 ms	RTS	10 B	Beacon	10 B
Contention time	10 ms	CTS	10 B	Contention period	8 slots
		SYNC	10 B	SIFS	48 μs

### 5.3. Performance Results

In this section, we present the performance results. At first, we discuss the emergency traffic. Then, we present results for combined traffic (normal and emergency). We also present a comparative performance study of the state-of-the-art protocols along with the proposed scheme.

#### 5.3.1. Emergency Traffic

In this section, the performance results for emergency traffic are presented. An emergency traffic generator is used to simulate emergency events. The rates of the events are increased from low to high. Our aim was to check possible scenarios ranging from a few emergency events per day to a large number of events reported by several BNs within a very short time. The results for energy consumption and delay are shown in Figure 17 and Figure 18, respectively. The number of BNs is varied from 1–30 for the evaluation.

The results show very promising trends. We can see that the energy consumption is reasonably low for such types of traffic. It increases as the number of nodes increases for obvious reasons. Even at a higher number of events, the energy consumption is still low. For 30 nodes and at a rate of 0.01, the energy consumption is only 0.05 J for the parameters presented in Table 4. It is slightly higher than the rate of 0.001. This shows that the proposed system can accommodate a large number of nodes at high event occurrences without significantly affecting the performance. Similarly, the delay results also show very encouraging trends. For 30 nodes, at a rate of 0.01, the delay is under 10 ms. At the lower rate, the delay is only 5 ms.

The results clearly show that the wake up radio system is able to inform the receiver about the current emergency event with low energy consumption and delay. The on-demand external wake up mechanism has helped to improve the performance. The sender does not need to wait for the awake time of the receiver node. A wake up radio enables prompt communication.

#### 5.3.2. Combined Traffic

In this section, we present the performance results for the combined traffic. As mentioned earlier, a BAN can have normal and emergency traffic. Now, our aim is to check the behavior of the wake up radio system when both normal and emergency traffic are present in the network. The normal traffic is generated through a predefined schedule for each BN. The schedule is maintained in a schedule table by the BNC. This helped to simulate the real-life scenario where the user or the doctor can assign a fixed packet transmission interval to each node. The emergency traffic is generated using a randomized traffic generator. The rate (packets per second) is varied from low to high. The network size is also varied with a maximum of 30 nodes. The metrics used are: energy consumption, delay and lifetime.

Figure 19 shows the energy consumption for the combined traffic. The results show lower energy consumption. In the proposed system, costly overheads, such as RTS and CTS, preambles and polling messages, which are commonly used in the traditional state-of-the-art MAC protocols, are reduced. Although the wake up radio process is similar to the RTS-CTS process, it does not use the main radio, unlike the traditional protocols. This helps to save energy in each transmission and reception. A wake up radio costs a mere few mW of power for transmission and few μW for reception of the packet. The proposed model is also able to maintain consistency in performance across a different number of nodes. For 20 nodes, at the rate of one packet per second, the energy consumption is under 0.5 J. This is reasonably low for such a system.

The delay performance for the combined traffic again shows encouraging trends as presented in Figure 20. For 10 nodes, the delay is under 130 ms when the rate is 0.01 packet/s. The delay increases with the increase in the rate and the number of nodes. For 30 nodes, the delay is still reasonably low (under 150 ms) when the rate is one packet/s. This shows the suitability of the proposed model for delay-sensitive BAN applications.

From the results, it is observed that low energy consumption is backed by lower delay for the combined traffic. The use of the wake up radio enables prompt communication. It is also able to avoid unnecessary wake up intervals. The lifetime is shown in Figure 21. The proposed MAC is able to achieve high lifetime due to lower energy consumption.

### 5.4. Comparative Study

In this section, we present the comparative performance evaluation of the proposed scheme with some popular and state-of-the-art MAC protocols. The aim is to highlight the merits of the wake up radio-based system compared to the existing protocols. The same setup is used for each of the protocols to normalize the results. At first, we present a comparison with the state-of-the-art non-standard MAC protocols. Then, we present a comparison with the current IEEE 802.15.x MAC protocols.

The state-of-the-art non-standardized MAC protocols considered for comparison are as follows:

- S-MAC;
- WiseMAC;
- T-MAC;
- X-MAC;
- B-MAC.

The standardized MAC protocols considered for comparison are as follows:

- IEEE802.15.4 MAC;
- IEEE802.15.6 MAC.

#### 5.4.1. Comparison with Non-Standard Protocols

**Figure 22:**
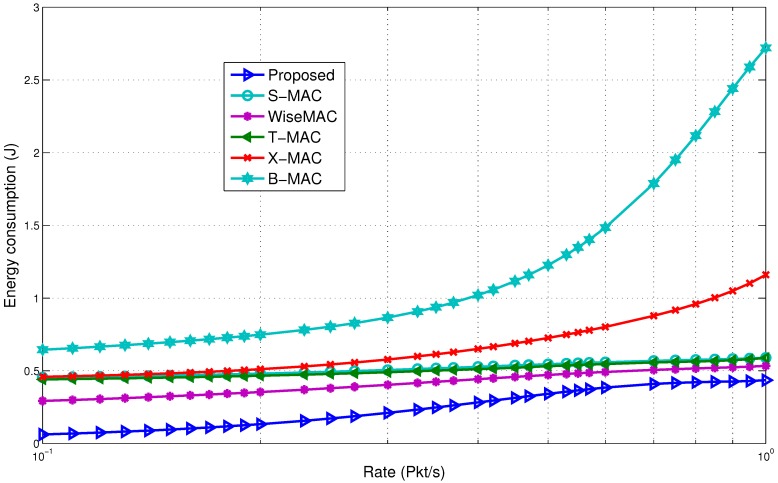
Energy consumption comparison with non-standard protocols.

The comparison for average energy consumption is shown in Figure 22. It is observed that the wake up radio system is able to improve the performance. At a low rate, the proposed model is able to save 3–5-times more energy compared to the rest of the protocols. The major reason is that the proposed model is able to eliminate the idle listening. It is one of the major causes of energy waste in a sensor network. In our model, a BN is able to avoid the unnecessary wake up periods. The proposed model is also able to avoid the periodic channel assessment and polling activities, which cause the extra use of energy in other protocols. There is also a difference in the data transfer initiation. Unlike the proposed model, the sender initiates the transfer in the rest of the MAC protocols. This may lead to missing the wake up time of the receiver, thus causing it to wait for a longer duration, which causes more energy consumption. This causes over-emitting, as the receiver is not ready, and the packet must be sent again.

The WiseMAC, B-MAC, T-MAC and X-MAC protocols are affected by the sampling period and traffic arrival rate. These protocols need to adjust their wake up period to optimize performance. B-MAC uses a long preamble, which causes it to use almost seven-times more energy than the proposed model. X-MAC is able to reduce the cost of the long preamble through the use of short strobe preambles. However, using two preambles on average for one successful data transmission again causes it to spend more energy. Reducing the overhead has helped the proposed model improve its overall performance. Use of the wake up radio has also reduced the overhearing energy consumption. For example, the RTS and CTS packets in the S-MAC protocol under the current scenario cause 39.5 mW of power to be used, while the proposed model uses around 2 mW of power. WiseMAC has the best results among the rest of the protocols. It adapts to the variable traffic. However, the preamble sampling still causes extra overhead energy consumption. In the rest of the MAC protocols, energy is also wasted due to idle listening. The proposed model is able to reduce the idle listening period, thereby saving energy, as explained in the previous section.

**Figure 23:**
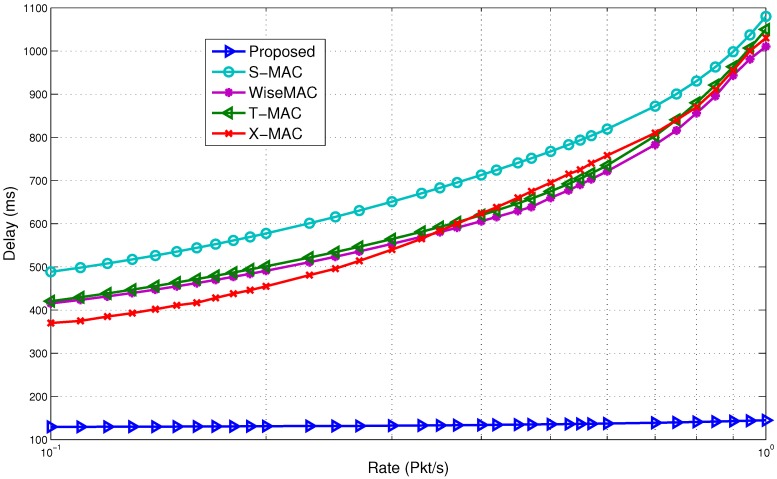
Delay comparison with non-standard protocols.

Figure 23 shows the delay comparison. It is observed that the proposed model outperforms the rest of the MAC protocols in terms of delay. At a low arrival rate, the delay is four-times lower for the proposed model. The results improve drastically as the rate goes up. A long wake up preamble causes a higher delay in WiseMAC. In the case of B-MAC, due to the long preamble, it requires extra time to transfer packets from the source to the destination. It has to send the entire preamble, even though the receiver is already awake. X-MAC acquires delay as the rate increases due to extra strobe preambles. Even though T-MAC tries to adapt to the traffic rate, it gains delay due to the timeout and early sleep problem. Once the awake timer expires, the receiver node goes to sleep mode even though the neighbor nodes want to send data to it. The sender has to wait until the receiver wakes up again, thus causing a longer delay. Due to these reasons, the gap between the proposed model and the rest of the protocols widens at the higher rate. The delay remains fairly consistent in the proposed model. This is evidently a major point to use the wake up radio-based system, as it supports prompt communication in a single hop network.

**Figure 24:**
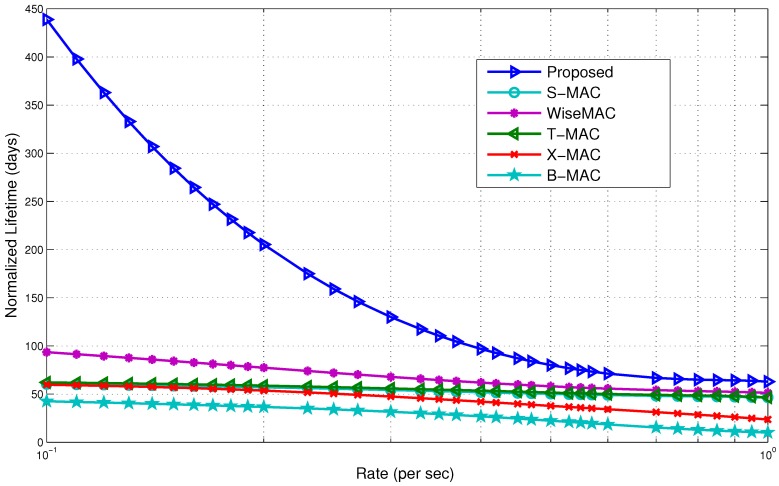
Lifetime comparison with non-standard protocols.

Figure 24 shows the lifetime comparison. The lifetime of the proposed model saw a significant increase. This is due to better conservation of energy compared to the rest of the protocols. At the low rate, the proposed model increased the lifetime by 4.5-times compared to WiseMAC. It outperforms B-MAC by a factor of nine at the low rate of 0.1. Our model is efficient in reducing potential causes of energy waste by avoiding beacons, poll messages, idle listening, periodic wake ups, *etc.* It is able to maximize the sleep time for BNs. The proposed model slightly outperforms other MAC protocols as the number of events increases. As the activities increased in the network, the lifetime also decreases and, finally, converges with the rest of the protocols at the high rate of 1 packet/s. This evidently shows the usability of the wake up radio system in a BAN.

#### 5.4.2. Comparison with Standard Protocols

Now, we present the performance comparison with two of the most well-known standard protocols that have been proposed and used for body-centric networks: the IEEE802.15.4 MAC and the IEEE802.15.6 BAN protocol. The purpose is to evaluate the performance of the wake up radio-based mechanism and the current standards. The packets are transmitted using the CSMA/CA mechanism in a superframe-based beacon-enabled mode. We have used the contention access period of the IEEE802.15.4 MAC as modeled in [35] and the exclusive access period (EAP1) of IEEE802.15.6 for the evaluation. The beacon-enabled superframe-based communication is used along with immediate acknowledgment for better reliability. The packet arrival rate is kept the same for all three protocols. We have simulated a scenario where several nodes try to send messages at the same time. This makes the arrival rate very high. We assumed 10 packets per second for the simulation. The proposed model uses the wake up radio system, and the standardized protocols use the CSMA/CA mechanism.

**Figure 25:**
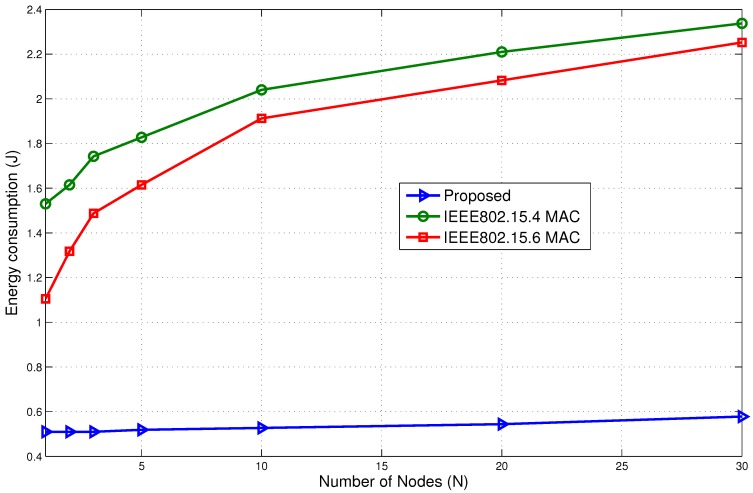
Energy consumption comparison with standard protocols.

Figure 25 shows the comparison of energy consumption. The proposed model is able to outperform the IEEE802.15.4 MAC and the IEEE802.15.6 MAC protocols. The high rate used for the comparison lowered the performance of the standard protocols. The results show that the lack of an immediate communication mechanism significantly affected the standard protocols. If the receiver is in the sleep state, the standard protocols are unable to communicate and, hence, have to wait until it is awake. This leads to unnecessary waiting time and higher energy consumption.

**Figure 26:**
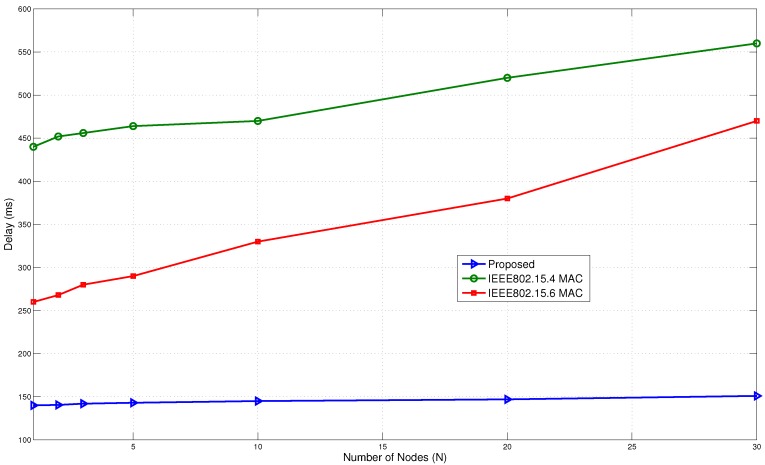
Delay comparison with standard protocols.

Figure 26 shows the delay comparison. The IEEE802.15.6 BAN performs reasonably well. It has a lower delay when the number of nodes is small in the network. However, as the number of nodes increases to 30, the delay also increases to around 450 ms. The IEEE802.15.4 MAC has the worst results due to the lack of a suitable immediate communication mechanism. With over 500 ms in delay, it is not suitable for several BAN applications.

**Figure 27:**
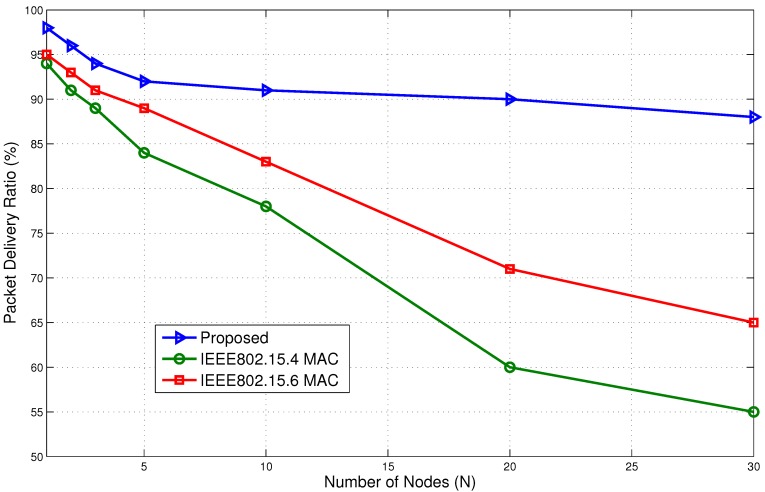
Packet delivery ratio (PDR) comparison with standard protocols.

The packet delivery ratio (PDR) is an important parameter. It is calculated as the ratio between the number of packets successfully received by the sink node and the actual number of packets sent by the nodes. The PDR is shown in Figure 27. The proposed model has slightly better PDR at the low number of nodes. It has 98% PDR compared to 95% for IEEE802.15.6 BAN and 94% for the IEEE802.15.4 MAC protocol. However, as the number of nodes increases, the PDR for IEEE802.15.4 decreases rapidly. The PDR for the IEEE802.15.4 MAC protocol reaches 55% compared to 65% for IEEE802.15.6 BAN and 88% for the proposed model. The proposed model is able to maintain a comparably higher success rate. The higher PDR is backed by the lower delay and energy consumption, which makes it a better choice for immediate packet communication for a BAN.

**Figure 28:**
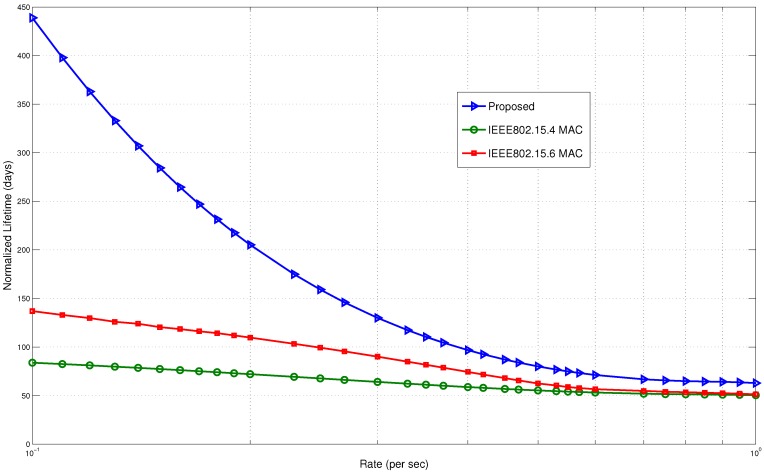
Lifetime comparison with standard protocols.

Finally, we present the lifetime comparison in Figure 28. The lifetime is normalized in terms of the number of days. It is observed that the proposed scheme outperforms both of these protocols. Following the trends in energy consumption, the proposed model has significant improvements in the lifetime. At the low arrival rate of 0.01 packets/s, the proposed model improved the lifetime by four times compared to IEEE802.15.4 MAC and three times compared to IEEE802.15.6 MAC. The wake up radio reduced the energy waste by minimizing the control packets. Unlike the standard protocols, it does not need to wait for the receiver to be awake for packet transmission. It enables prompt delivery of packets, thereby saving energy.

## 6. Sensitivity Analysis

In this section, we present a discussion of the impact of factors, such as overheads and packet size, on the existing and proposed schemes. We also present a component-wise analysis for energy consumption and the wake up time and overhead times of the discussed systems.

### 6.1. Impact of Overhead on Energy Consumption

**Figure 29:**
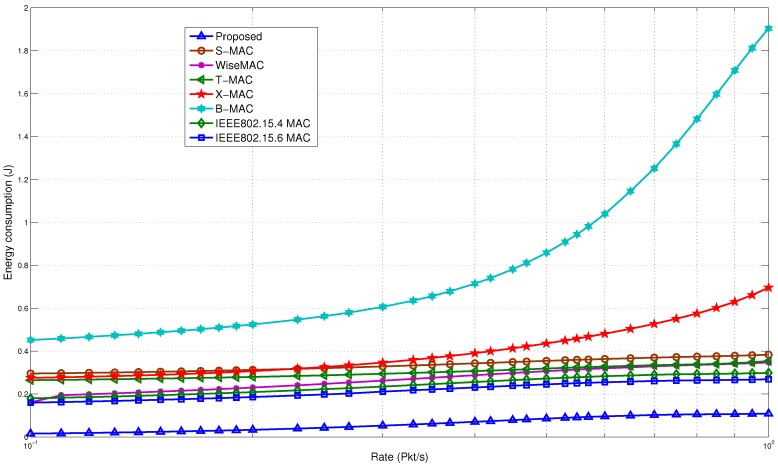
Impact of overhead on energy consumption.

The overheads affect the performance of a MAC protocol. It is a major design factor. MAC protocols, such as B-MAC and X-MAC, have reduced performance due to the energy consumed by overheads. Therefore, reducing the overheads is one of our major design goals. Our aim was to reduce overheads as much as possible. The impact of overhead on energy consumption is shown in Figure 29. B-MAC has the largest overheads due to the long preamble, which significantly increases the energy consumption as the traffic rate becomes higher. X-MAC improves on the results of B-MAC through the use of small strobe preambles. T-MAC, S-MAC and WiseMAC have a lower proportion of overheads than B-MAC and X-MAC. However, they are still a bigger part in the total energy consumption. The RTS-CTS packet handshaking is a major cause of energy consumption. Since these protocols use the main radio to send the control packets, the energy consumption is very high. The transmission and reception power of 26 mW and 13.5 mW affects every control packet. As the number of nodes increases with a higher arrival rate, the energy consumption also increases. The same handshake is performed using the wake up radio in the proposed model, which uses very low power for operation (in μW). The standard IEEE802.15.4 MAC has to contend with two clear channel assessments (CCA) before attempting any transmission. The IEEE802.15.6 MAC protocol suffers from typical CSMA/CA-based overheads along with beacon packets. The overhead energy consumption is still higher than the proposed model.

As mentioned earlier, it is a common practice to turn off the main radio to save energy. However, it is very difficult to manage the wake up schedule in an energy-efficient way. A wake up radio system can easily avoid the periodic wake ups of the schedule-based MAC protocols. The nodes in these protocols have to wake up even though there are no packets to receive. Similarly, a wake up radio system can avoid the periodic channel assessment. It can also avoid idle listening, which is done in anticipation of new packets. The use of a wake up radio also reduces energy consumption due to control packets in the network.

### 6.2. Impact of Overhead on Delay

**Figure 30:**
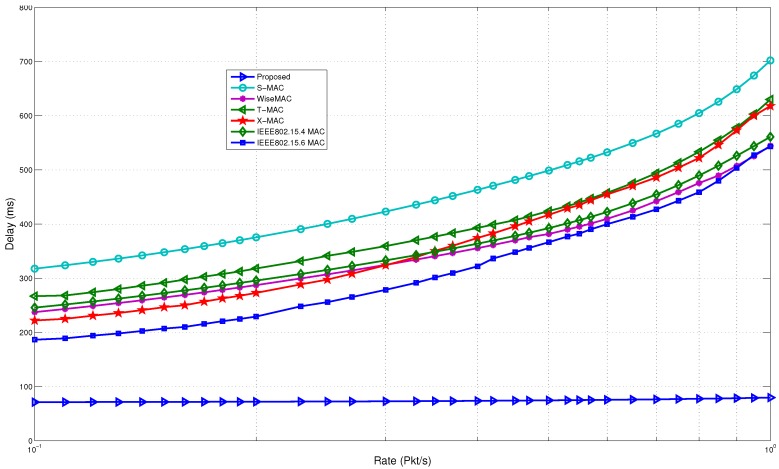
Impact of overhead on delay.

The overheads also affect the delay. The most affected is the S-MAC protocol. It has a large number of control packet overheads in the network. The synchronization packet and the RTS-CTS packets cause higher delay. It also has to deal with fixed wake up periods, which causes a longer waiting time for the sender. The impact of overhead on delay is shown in Figure 30. WiseMAC has to deal with a long preamble. X-MAC incurs overheads from multiple strobe preambles. The IEEE802.15.x MAC protocols have channel assessment and control packet delay overheads. The proposed MAC gets overheads from the wake up radio. However, on-demand and efficient scheduling has reduced overall overheads in the network. On average, it is able to decrease overhead delay by 2–3 times compared to the rest of the protocols. The wake up radio also reduces energy consumption by reducing the waiting time. It maintains prompt and immediate communication for emergency packets.

### 6.3. Impact of Packet Size

The impact of the data packet size is homogeneous for all the MAC protocols, as is evident from the energy consumption and delay expressions. However, changing the size of the control packets used in scheduled and unscheduled MAC protocols can individually affect their performance. For example, reducing the preamble size in B-MAC can reduce the delay, while increasing the miss probability. The smaller preamble in X-MAC can lower delay, as well. The increase in the length of the control packets in WiseMAC does not affect its performance. This is also true for the IEEE802.15.x MAC protocols.

### 6.4. Component-Wise Analysis

The component-wise energy consumption is presented in Table 6. The main components are wake up, transmit, receive and overheads. The parameters used are presented in Table 4 and Table 5. All units are in Jules. We can clearly see that the proposed system is ahead in terms of energy conservation. The data and acknowledgment (ACK) packets are transmitted using the main radio in the case of all of the systems. However, the wake up process is handled by the wake up radio for the proposed system, preamble sampling for WiseMAC, beacon for the IEEE802.15.4x protocols, RTS/CTS for the S-MAC and T-MAC protocols, long preamble for B-MAC and short strobe preambles for the X-MAC protocol. The proposed protocol is able to reduce the overheads significantly. It contributed to the overall reduction in the energy consumption for the proposed scheme.

**Table 6:** Component-wise break down of energy consumption (units are in Joules).

Components	Proposed	WiseMAC	X-MAC	B-MAC	T-MAC	S-MAC	IEEE802.15.4	IEEE802.15.6
Wake up/Preamble	0.0046	0.246	0.211	0.213	0.472	0.55	0.968	0.557
Transmit data	0.3936	0.3936	0.492	0.984	0.394	0.472	1.26	1.26
Receive ACK	0.0768	0.0768	0.0768	0.0768	0.0768	0.0768	0.0768	0.0768
Overheads	0.015	0.162	0.296	0.274	0.265	0.205	0.179	0.158

The wake up time and overhead times of the discussed systems are presented in Table 7. All units are in milliseconds. It is evident from the table data that the proposed system spends the least amount of time before completing the communication process. Thus, the proposed scheme saves much time. It does not waste time in unnecessary wake up times, which causes it to save energy and also reduce the delay. Therefore, the wake up radio system shows great potential for a BAN. It has the added advantage of communicating with a sleeping node as and when necessary. Thus, emergency communication can be promptly handled.

**Table 7:** Break down of wake up time and overhead time (units are in milliseconds).

Components	Proposed	WiseMAC	X-MAC	B-MAC	T-MAC	S-MAC	IEEE802.15.4	IEEE802.15.6
Wake up	0.802	20	5.61	86.7	64	96	40	20
Overheads	72.1	238	223	240	260	302	290	187

## 7. Conclusions

Sensor devices to monitor human body functions are being developed. The body area network has tremendous growth potential. In this paper, we present an out-of-band wake up radio scheme to manage packet transmission in a BAN. It uses an external on-demand mechanism to wake up a sleeping node for communication. The motivation behind our work is that a node should only wake up when it needs to receive or transmit a packet. Over-emitting, idle listening and overhearing cause a significant amount of energy to be wasted in traditional state-of-the-art protocols. A wake up radio system can reduce such overheads through the on-demand process. Using a suitable out-of-band wake up, energy can be saved and thereby increasing the lifetime of the nodes. It is also observed that existing schemes are not sufficient enough to handle emergency traffic in a BAN. A wake up radio-based system supports prompt communication. It can reduce the overheads’ energy consumption. It also minimizes the need of the unnecessary wake up time for the receiver nodes. The proposed scheme has better performance with the added advantage of the ability to communicate with a sleeping node. It reduces the waiting time of the receiver. Our future works include the design and analysis of a robust and secure wake up mechanism for a BAN.
